# Supplementary material for: Development of a computer-aided design software for the quantitative evaluation of aesthetic damage
Source: PLoS One. 2019 Dec 18;14(12):e0226322. doi: 10.1371/journal.pone.0226322 (PMC6919621; doi:10.1371/journal.pone.0226322)
Supplement: S1 Code site — 1. (ZIP) [file pone.0226322.s001.zip › [EESC_jr][Projeto]Relatorio_07_21/vendors/select2/docs/_includes/options-old/events.html]

# Events

Select2 has an internal event system that is used to notify parts of the
component that state has changed, as well as an adapter that allows some
of these events to be relayed to the outside word.

Adapter
:   `SelectionAdapter`

Decorator
:   `EventRelay`

## Public events

All public events are relayed using the jQuery event system, and they are
triggered on the `<select>` element that Select2 is
attached to. You can attach to them using the
`.on` method provided
by jQuery.

## Internal events

Select2 triggers internal events using its own internal event system,
which allows adapters to communicate with each other. These events are not
accessible through the jQuery event system.

You can find more information on the public events triggered by individual
adapters in the individual adapter documentation.
